# Evaluating the semi-chronic effects of household air pollution exposure on cardiopulmonary health under two different ventilation conditions

**DOI:** 10.1038/s41598-025-29728-2

**Published:** 2026-03-28

**Authors:** Sara Aristizabal, Eric M. Snyder, Zachary C. Pope, Araliya M. Senerat, Kunjoon Byun, Qingyang Liu, Young Joo Son, Linhao Li, Aidan Mullan, Bruce D. Johnson, Veronique Roger, Nicholas Clements, Jovan Pantelic

**Affiliations:** 1 Delos Living LLC, New York, NY USA; 2 Well Living Lab, Rochester, MN USA; 3 TSET Health Promotion Research Center, Stephenson Cancer Center, University of Oklahoma Health Sciences, Oklahoma City, USA; 4 Department of Health Promotion Sciences, Hudson College of Public Health, University of Oklahoma Health Sciences, Oklahoma City, USA; 5 Phipps Neighborhoods, New York, NY USA; 6 Georgia Institute of Technology, Atlanta, GA USA; 7 PAXAFE, Inc, Milwaukee, WI USA; 8 Department of Quantitative Health Sciences, Mayo Clinic, Rochester, MN USA; 9 Division of Preventive Cardiology, Mayo Clinic, Rochester, MN USA; 10 National, Heart Lung and Blood Institute, Bethesda, MD USA; 11 University of Colorado, Boulder, Boulder USA

**Keywords:** Environmental sciences, Health care, Medical research, Risk factors

## Abstract

Household air pollution (HAP), particularly from cooking-related particulate matter (PM_2.5_), poses significant health risks but remains understudied compared to ambient air pollution. We evaluated the short-term cardiorespiratory effects of exposure to cooking-generated PM_2.5_ and examined the efficacy of automated indoor air quality interventions. Using a crossover design, seven cohorts of two participants each were exposed to two residential conditions over four weeks in a Living Lab: the Standard Control Condition (SCC), featuring basic HVAC, and the Advanced Control Condition (ACC), which included automated range hoods, portable air cleaners and exhaust systems activated by PM_2.5_ sensors. PM_2.5_ concentrations were continuously monitored in the breathing zone at the room level. The physiological markers, blood pressure (BP), heart rate (HR), heart rate variability (HRV) and fractional exhaled nitric oxide (FeNO), were measured on the occupant before and after cooking events. Cooking events caused substantial short-term increases in PM_2.5_ levels, median concentrations rose from < 1 µg/m³ to 263.7 µg/m³ under SCC and to 168.9 µg/m³ under ACC during HRV measurement periods, with exposure levels exceeding WHO 24-hour guidelines up to 82% of the time. Compared to SCC, the ACC significantly reduced PM_2.5_ exposure (*p* < 0.05). Systolic blood pressure (SBP) decreased significantly post-cooking under ACC (ΔSBP = −3.1 ± 10.0 mmHg) but not in the SCC (ΔSBP = −0.9 ± 8.0 mmHg; *p* < 0.05). HR and HRV showed no statistically significant differences between conditions, though trends in RMSSD, SDNN and LF/HF ratio suggested improved autonomic balance under ACC. HR decreased post-cooking under ACC but increased slightly under SCC (ΔHR = −4.5 ± 6.5 bpm vs. 1.0 ± 1.1 bpm; 95% CI: (−9.8 to −1.2)). FeNO decreased significantly within both conditions pre- to post-cooking, but the difference in reduction between conditions did not reach statistical significance, despite a trend toward greater decline in the ACC. These findings suggest that semi-chronic exposure to cooking-related PM_2.5_ can adversely affect cardiovascular function, particularly systolic BP and HR, and that automated indoor air quality interventions can meaningfully reduce pollutant exposure and associated physiological impacts. Our results support the implementation of HAP mitigation strategies in residential settings and highlight the need for further research among populations with existing cardiopulmonary conditions.

## Introduction

Air pollution remains one of the most significant public health and environmental challenges worldwide^1^. It ranks as the fourth leading risk factor for disease burden, contributing approximately 200 million disability-adjusted life years (DALYs) and nearly 6.67 million deaths annually^1^.

Exposure to air pollution occurs in both ambient (outdoor) and indoor environments such as homes and workplaces. Among pollutants, fine particulate matter (PM_2.5_) is particularly harmful. Exposure to ambient PM_2.5_ pollution has been linked to a range of cardiovascular outcomes including cardiac arrhythmias, hypertension, atherosclerosis, ischemic stroke and myocardial infarction^2^. Similarly, both ambient and indoor PM_2.5_ exposures affect respiratory health, contributing to asthma, chronic bronchitis, acute respiratory infections, impaired lung function and chronic obstructive pulmonary disease (COPD)^3^. Short-term increases in PM_2.5_ also impact autonomic nervous system balance by reducing heart rate variability and causing fluctuations in blood pressure, as well as triggering airway inflammation and hyperreactivity^4,5^. These effects can induce or exacerbate cardiovascular and respiratory diseases^6^.

Ambient PM_2.5_ primarily originates from vehicle emissions, industrial processes and the combustion of household fuels, along with other anthropogenic activities^7^. Indoors, PM_2.5_ can originate from both infiltrated outdoor air and various indoor activities, most notably cooking and heating^8^. Given that individuals spend over 90% of their time indoors^9^, indoor sources can contribute to between 19% and 76% of a person’s total PM_2.5_ exposure^10^. Although indoor PM_2.5_ exposure accounts in many cases for majority of the overall daily aerosol exposures, the impact of indoor PM_2.5_ on health has been studied less extensively^11^, due to complexities like variable indoor environments and diverse emission activities^12,13^.

Among indoor sources, cooking is considered one of the most significant contributors to PM_2.5_ levels^11^. Studies have shown that cooking activities can elevate indoor PM_2.5_ concentrations to levels several times higher than the daily thresholds suggested by indoor air quality standards^14^. The quantity and composition of emitted particles vary depending on factors such as the cooking method, temperature and type of food^15^, yet the health impacts remain consistent, cooking activities have been demonstrated to negatively impact cardiovascular and respiratory health^16^. Additionally, cooking releases a variety of inorganic and organic compounds, including polycyclic aromatic hydrocarbons, many of which are known or suspected carcinogens^17^.

Dispersion patterns of cooking-generated particles suggest that adverse health effects associated with the exposure to cooking emissions are not limited to occupants in the kitchen^16–18^. For instance, Wan et al.^17^ observed that cooking increased ultrafine particle (UFP) concentrations in the living room, located away from the kitchen, by up to 2.7 times above baseline levels.

Considering the absence of universally safe exposure thresholds for indoor air pollutants and the variability of guideline recommendations, controlling emission sources within indoor environments remains critical^19^. Indoor PM_2.5_ can be mitigated through several methods that include natural ventilation^20^, stove hoods^19^, portable air cleaners (PACs)^21^, enhanced mechanical ventilation^21^ or a combination of these approaches^18^. Stove hoods are the most effective means to manage cooking emissions^22^, but their effectiveness in reducing exposure is limited by low average usage given the necessity to turn them on manually^23,24^. PACs offer the flexibility to be used in various indoor spaces without requiring permanent installation or extensive renovations. With automated PM-sensor operation, they also achieve good effectiveness in capturing a wide range of airborne particles^25^. However, their effectiveness can vary based on the specific filtration technology and the occupants’ usage patterns, including the airflow settings and the duration the device is operational^25^. Regular maintenance, such as filter changes, is also essential to maintain efficiency^25^. Additionally, placement is important, positioning the PAC closer to the emission source can improve its ability to reduce PM concentrations more rapidly^26^.

We evaluated the effects of a set of automated air quality improvement interventions on cardiovascular and respiratory health in healthy individuals exposed to cooking-induced particulate matter. We assessed cardiorespiratory function under two different conditions: a Standard Control Condition (SCC), which replicated standard house HVAC operation as described in ASHRAE Standard 62.2–2019^27^, and an Advanced Control Condition (ACC), which included automated range hoods, PACs and exhaust systems operated based on the PM_2.5_ readings from the sensors strategically placed in the kitchen, bedroom, living room and bathroom of two small apartments within a Living Lab setting.

Seven cohorts of two participants experienced each condition for two weeks, totaling four consecutive weeks of Living Lab occupancy. Comparisons were made between short-term monitoring of physiological indices of cardiorespiratory health using non-intrusive devices—heart rate (HR), heart rate variability (HRV), blood pressure (BP) and exhaled nitric oxide (FeNO)—across the two ventilation conditions, SCC and ACC.

We hypothesized that the ACC would better mitigate potential cardiorespiratory effects and effectively reduce PM dispersed across the residential environment relative to the SCC.

## Results

### Participant flow

**Fig. 1:**
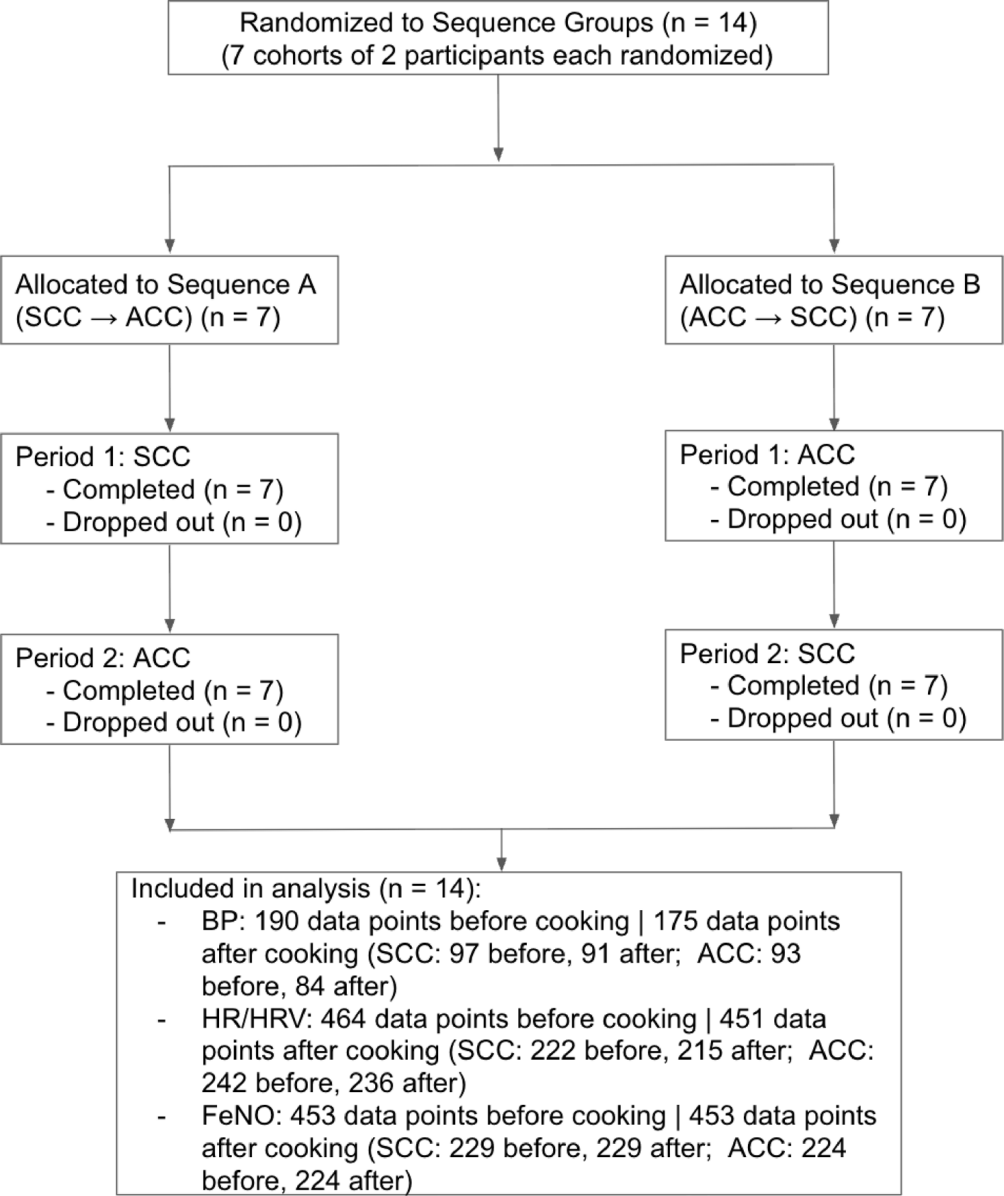
CONSORT flow diagram depicting the progression of participants through the study phases, including recruitment, randomization, allocation to experimental condition sequences and inclusion in the final analysis.

Figure 1 illustrates the flow of participants through the study, including recruitment, randomization, exclusions and inclusion in the final analysis. The diagram also shows the distribution of participants across the different condition sequences and period interactions.

### PM_2.5_ concentrations

**Fig. 2:**
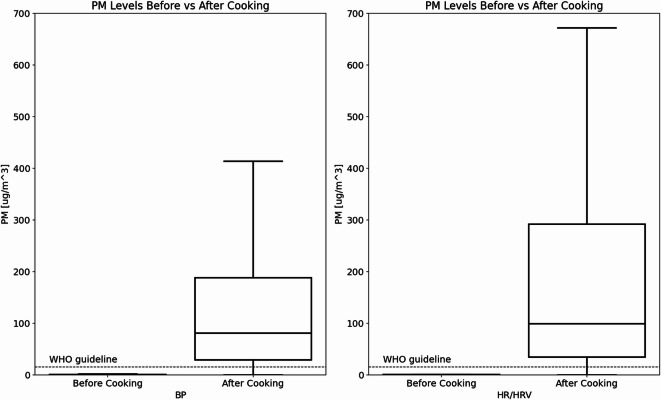
PM_2.5_ levels before and after cooking started, along with: blood pressure (BP) and heart rate/heart rate variability (HR/HRV) measurements. In comparison to the WHO 24-h guidelines, which suggest a PM_2.5_ concentration of 15 µg/m³ (blue dashed line).

**Fig. 3:**
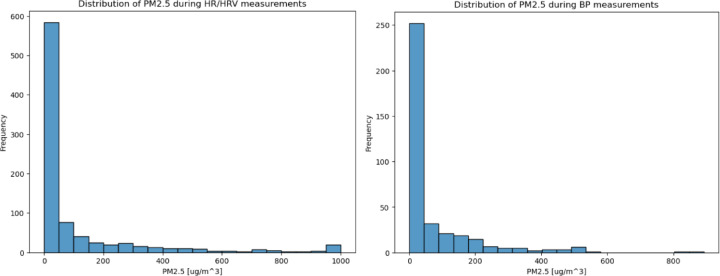
Frequency distribution of PM_2.5_ during HR/HRV and BP measurements.

When PM_2.5_ measurements were filtered for periods during BP measurement and HRV measurement (Fig. 2), the combined data for the SCC and ACC demonstrates that the median PM_2.5_ concentration before cooking was below the sensor detection limit of 6 µg/m³. This result suggests that study participants were inhaling clean indoor air before they started to cook. In other words, during the baseline period before cooking (for the BP measurements), PM_2.5_ concentrations remained below the WHO 24-hour guideline of 15 µg/m³ throughout the entire measurement period^28^. During the HRV measurements, however, there were several short-term increases in PM_2.5_ concentrations above 15 µg/m³, resulting in levels staying below the WHO 24-hour guideline for 41% of the time. The distribution of concentrations can be observed in Fig. 3, which shows the frequency of different PM_2.5_ levels. The results indicate that the majority of the measurements were below 25 µg/m³. At the time of the BP measurements, the median PM_2.5_ concentration had risen to 135.7 µg/m³ after cooking started, representing a significant pre- to post-cooking increase (t = 11.93, df = 182.02, p-value < 0.001). These results also show that PM_2.5_ levels exceeded the WHO 24-hour guideline 40% of the time, as illustrated in Fig. 3. At the time of HR/HRV measurements, PM_2.5_ levels increased to 213.5 µg/m³ after cooking started, a significant pre- to post-cooking increase (t = 16.864, df = 430.25, p-value < 0.001). These results suggest that PM_2.5_ levels were above the WHO 24-h guideline for 82% of the time. Cooking resulted in short-term PM_2.5_ increases that were approximately two orders of magnitude above the baseline concentration.

**Fig. 4:**
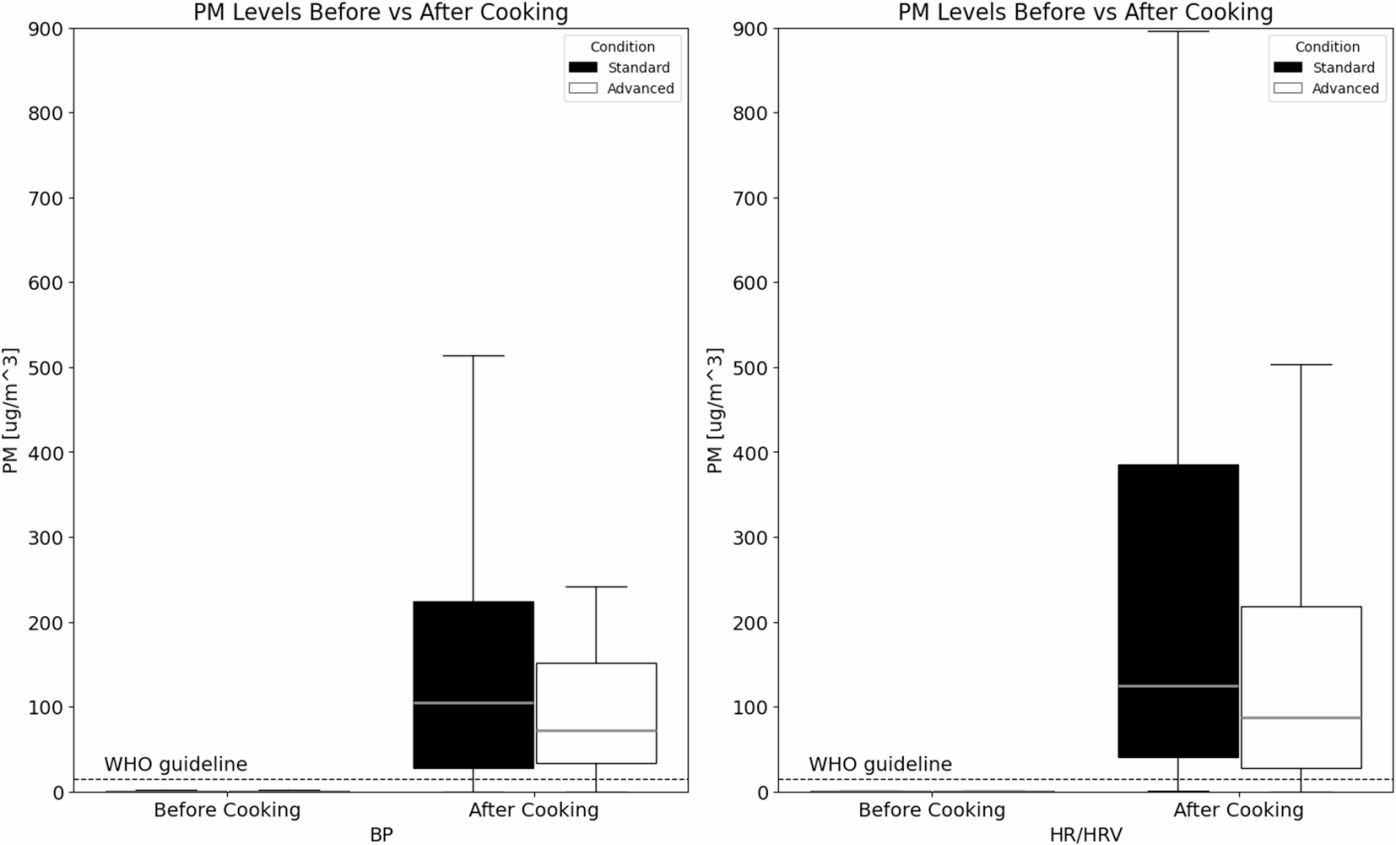
PM_2.5_ levels before and after cooking started for: (**a**) BP and (**b**) HR/HRV measurements. In comparison to the WHO 24-h guidelines, which suggest PM_2.5_ concentrations should not exceed 15 µg/m³.

When PM_2.5_ measurements were further categorized by HR/HRV and BP groups, and then subdivided into SCC and ACC groups (Fig. 4), results indicated that the median for the SCC PM_2.5_ concentration before cooking was 0.69 µg/m³ and the median after cooking started was 263.7 µg/m³ during HR/HRV measurements. During the ACC, the mean PM_2.5_ concentration before cooking was 0.95 µg/m³ and increased to 168.87 µg/m³ after cooking during HR/HRV measurements. This difference between the SCC and ACC was statistically significant (t = −3.75, *p* < 0.05) and suggested the need to examine HR/HRV differences between the two conditions, as well as periods before and after cooking started. A different trend was observed during the periods when BP measurements were taken. Before cooking, results suggest that PM_2.5_ levels were below the detection limits for both conditions. After cooking started, there were no statistically significant differences between the SCC and ACC (t = −1.25, *p* > 0.05), suggesting that the environmental conditions were not significantly different during the BP measurement periods. As a result, comparisons between the SCC and ACC would not be informative. However, the results do support the feasibility of examining BP measurements before and after cooking, as there are statistically significant changes in environmental conditions over time.

### Heart rate and heart rate variability

Initially, correlations were performed on all data points to explore the relationship between PM_2.5_ and both HR and HRV under each condition (before cooking, after cooking, before and after cooking just during SCC and before and after cooking during ACC). When data from both conditions (SCC and ACC combined) were analyzed, a small but significant relationship was observed between PM_2.5_ and HR both before and after cooking (HR before: *r* = 0.12, *p* < 0.05; HR after: *r* = 0.15, *p* < 0.05). No significant correlations were found between PM_2.5_ and any HRV parameters in either time window.

Further analysis showed that following cooking under the SCC condition, but not under ACC, there was a significant positive correlation between PM_2.5_ and HR (*r* = 0.22, *p* < 0.01). Again, no significant associations were found between PM_2.5_ and HRV parameters within either condition.

**Fig. 5:**
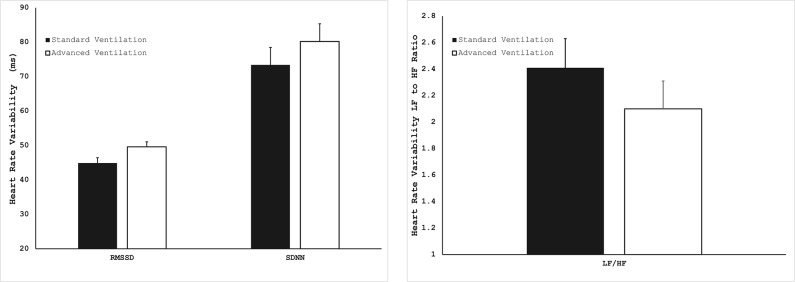
Heart rate variability parameters (RMSSD, SDNN and LF/HF ratio) measured upon waking.

A summary of HRV measurements obtained across participants is shown in Table 1; Fig. 5. There were no significant differences in absolute measures of HR or HRV or in the percent change in HRV time- and frequency domain parameters. Although the root of the mean square of successive differences (RMSSD) and the standard deviation of normal-to-normal intervals (SDNN) were higher in the ACC and the ratio of low-frequency to high-frequency power (LF/HF) was slightly lower (indicating slightly higher parasympathetic nervous system activity), none of these parameters reached statistical significance.

**Table 1:**
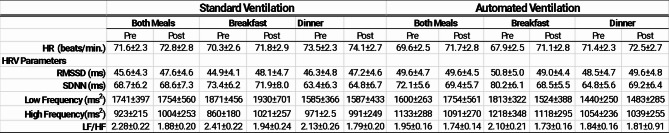
Heart rate variability parameters with each ventilation condition before and after cooking.

The change in HR and HRV parameters from pre- to post-cooking was also assessed under both SCC and ACC conditions. HR and the LF/HF ratio were found to differ significantly between ventilation conditions (ΔHR = −4.5 ± 6.5 bpm for ACC vs. 1.0 ± 1.1 bpm for SCC; 95% CI: (−9.8 to −1.2)) and (ΔLF/HF = 0.15 ± 0.71 for ACC vs. −0.38 for SCC; 95% CI: (0.07 to 1.01)).

### Blood pressure

**Fig. 6:**
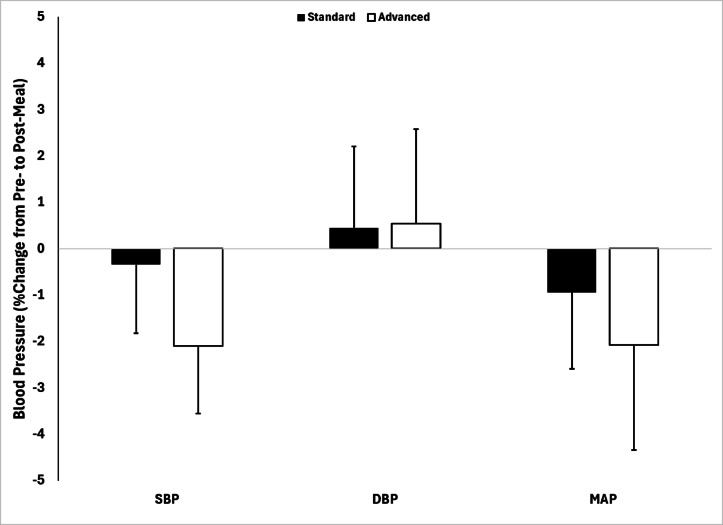
Blood pressure percent change with condition from before to after cooking with both meals.

Initially, correlations were performed on all data points to explore the relationship between PM_2.5_ and SBP, DBP and MAP under each condition (before cooking, after cooking, before and after cooking just during SCC and before and after cooking during ACC). When considering both conditions together (SCC and ACC), no significant relationships were observed between PM_2.5_ levels and any BP measurements before cooking. However, a small but statistically significant correlation was found between PM_2.5_ levels and MAP after cooking (*r* = 0.20, *p* < 0.01). When analyzed by condition, no significant correlations were observed between PM_2.5_ and any BP measures after cooking under SCC. In contrast, under ACC, a small but statistically significant relationship was found between PM_2.5_ and MAP following cooking (*r* = 0.25, *p* < 0.05). The results from the BP variables are presented in Table 2; Fig. 6.

**Table 2:**
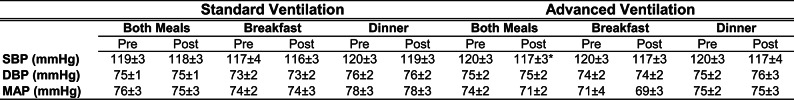
Blood pressure parameters before and after cooking under each condition.

We found a significant effect of condition for BP when examining systolic blood pressure (SBP; *p* < 0.05), but not diastolic blood pressure (DBP) or mean arterial pressure (MAP). Specifically, SBP decreased post-cooking in the ACC, but not in the SCC (ΔSBP = −3.1 ± 10.0 mmHg vs. −0.90 ± 8.0mmHg; 95% CI: (8.2, 2.0); Changes in DBP and MAP were not statistically significant between conditions (ΔDBP = −0.07 ± 8.0mmHg vs. 0.07 ± 7 mmHg; 95% CI: (−2.8 to 2.8); ΔMAP = −3.0 ± 10.9 mmHg vs. −0.88 ± 8.2 mmHg, for ACC and SCC, respectively). Although a similar downward trend in SBP was observed when expressed as a percent change from pre-cooking, this difference did not reach statistical significance.

### Estimate of lung function (Fractional exhalation of nitric Oxide, FeNO)

**Fig. 7:**
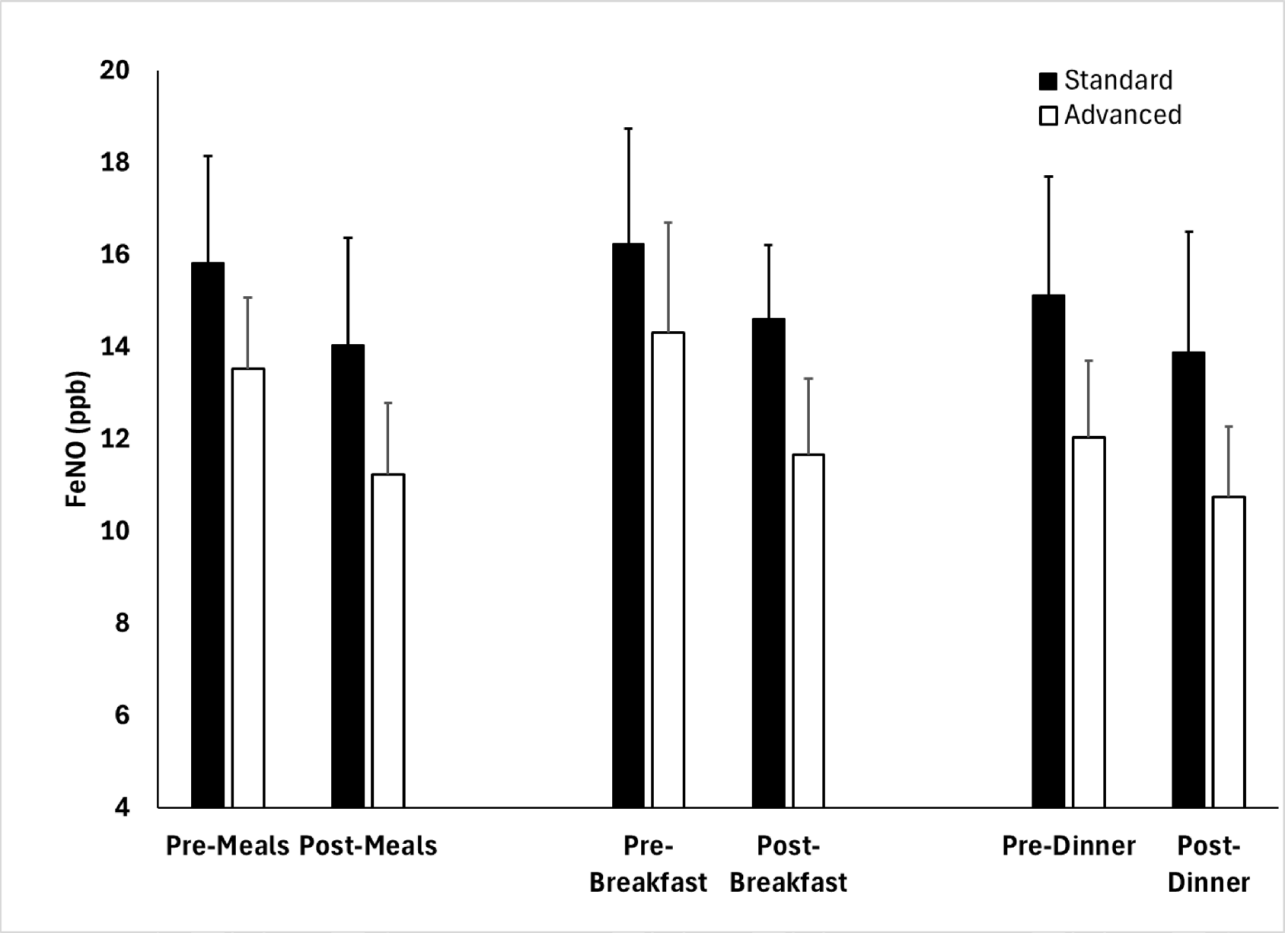
FeNO under each condition from before to after cooking.

**Fig. 8:**
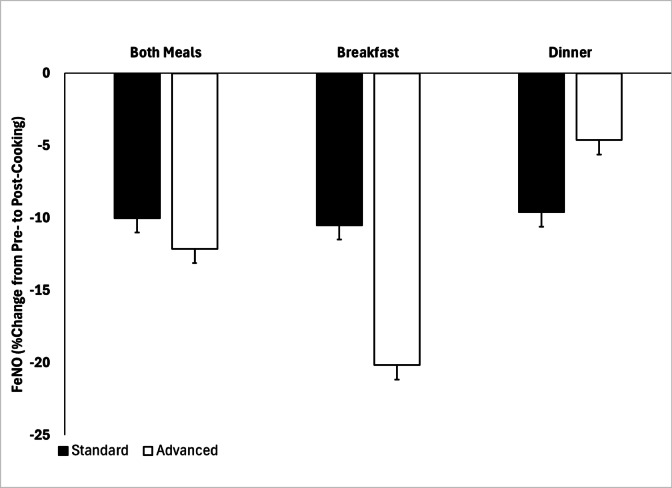
FeNO percent change with condition from before to after cooking with both meals.

A summary of FeNO measurements obtained across participants is shown in Fig. 7. We observed median FeNO values in the SCC of 14.0 ppb (IQR 8.0–22.7) and 10.7 ppb (6.7–19.3) pre- and post- cooking, respectively, with median FeNO values in the ACC being 13.2 ppb (IQR 8.1–17.7) and 10.2 (IQR 6.0–15.3) during these time segments. Trends in mean values of FeNO were similar. In fully-adjusted generalized estimating equations (GEE) models, we observed within-condition reductions in FeNO values pre- to post-cooking to be significant during both conditions (SCC: β = −2.30, 95% CI: (−3.44 to −1.16), *p* < 0.001; ACC: β = −2.79, 95% CI: (−4.21 to −1.36), *p* < 0.001), but these reductions were not significantly different between conditions. Expressed as a percent change from pre- to post-cooking values (Fig. 8), there was a trend towards a greater decrease post-cooking in the ACC, particularly during breakfast, but this did not reach statistical significance (*p* = 0.13).

There was a significant difference in the change in FeNO between ACC and SCC following cooking exposure (ΔFeNO = − 1.7 ± 5.8 ppb vs. 3.0 ± 3.7 ppb for ACC and SCC, respectively; 95% CI: (0.9, 8.2)).

## Discussion

We sought to evaluate whether short-term (weeks) differential exposure to cooking-generated particles, based on ventilation control conditions, had a physiological effect on cardiorespiratory outcomes. Exposure to ambient PM has been associated with increased cardiovascular and respiratory disease risk, with exposure to ambient PM_2.5_ linked with increased SBP, HRV changes and decreased pulmonary function^4,29,30^. While the Global Burden of Disease Study has stated that nine million deaths occur globally each year due to air pollution^31^, it is worth noting that 3.2 million of these deaths have been attributable to household air pollution (HAP)^32^. However, few studies have examined the effects of HAP, and cooking activities specifically, on occupant health and well-being. This renders the observations during the current investigation particularly important.

We observed the ACC to result in greater SBP reductions pre- to post-cooking than the SCC. This aligns with previous work that has demonstrated an effect of PM exposure on both acute and chronic BP changes^33,34^. Interestingly, this was a healthy population with an average baseline SBP of < 120mmHg. We would anticipate a much larger effect on BP in a hypertensive population. If the trend were to hold in a hypertensive population, we would anticipate the magnitude of drop in BP with the ACC to be larger than 5mmHg, which the American Heart Association has considered clinically meaningful^35^. It is possible that this magnitude of reduction is large enough to have a protective effect on the cardiovascular system. Although differences in BP between the ventilation conditions were observed in the present study, and BP values were slightly lower during periods when average PM_2.5_ levels were also lower over the two week period, the primarily aim of this study was not to assess the acute effects of PM_2.5_ on the cardiopulmonary response or to establish a dose-response relationship between PM_2.5_ and these outcome variables. The findings suggest a potential effect of the two ventilation strategies on PM_2.5_ and cardiopulmonary function over time; however, these should be considered preliminary. Future studies with larger sample sizes and adequate statistical power should focus on these outcomes within narrowly defined time windows and incorporate more granular PM_2.5_ measurements to better evaluate potential dose-response relationships.

We did not observe the ACC to result in any meaningful changes in time- and frequency-domain HRV. There were two potentially confounding variables that affected the HRV measures in the present study. First, participants took their initial HRV measure upon waking (which was up to three hours before cooking in some individuals). Second, while HRV measurements taken at breakfast were likely unaffected by prior food intake — due to the natural fasting state following sleep — dinner measurements may have been influenced by eating beforehand (e.g., snacking), as previous research has shown that food intake can affect HRV, likely due to the shift toward parasympathetic activity during digestion^36^; In addition, meal composition may also impact HRV variables^37^. Given the relatively small sample size in the present study, these two potential confounding factors could have influenced the results.

Although significant reductions in FeNO values were observed pre- to post-cooking in both conditions, there were no differences between conditions. Although not significant, we did find that the ACC led to a greater reduction in FeNO, when compared to the SCC. This difference was most prominent during the breakfast measure, which may indicate that there are less statistical confounding parameters, such as the participants leaving the control environment during the day, which may be affecting pollution exposure and the dinner-time measures of FeNO. Also of interest is that the baseline FeNO values were very low in this group and within the healthy and normal range. Participants with healthy airways may not exhibit as large of an effect of lung function variables following an intervention, when compared to patients with airways disease (i.e. asthma, COPD)^38^. Similar to our findings in BP, we would anticipate an augmented effect of these FeNO differences in patients in asthma or COPD, which could be a particularly exciting area of study.

Particulate air pollution can promote systemic inflammation and oxidative stress. Common household activities, such as cooking and cleaning, emit indoor air pollutants (e.g., PM_2.5_, PM_10_) that act upon select inflammatory pathways and have detrimental impacts on cardiopulmonary outcomes^16,39,40^. Although we only observed trends by condition for HRV and FeNO parameters, we did observe a significant impact on BP. Two primary pathways may explain our observations. First, PMs may activate pro-inflammatory macrophages in the lungs, releasing pro-inflammatory cytokines^41,42^. In circulation, pro-inflammatory cytokines promote arterial stiffness, increased BP, and, in some cases, thrombosis^43^. A second pathway links PM exposure to autonomic nervous system (ANS) sympathetic-parasympathetic activity.

In this pathway, PMs activate alveolar and neurological receptors, causing adverse changes in ANS sympathetic-parasympathetic output, detrimental to cardiac autonomic functioning and observed as lower time-domain HRV metrics and higher LF power band activity in the frequency domain. Further, the parasympathetic branch of the ANS is important in downregulation of pro-inflammatory cytokines^44^. Thus, as cytokines are upregulated by repeated exposure to PMs and other air pollutants, decreased parasympathetic output may allow pro-inflammatory cytokines to circulate unabated, promoting low-grade microvascular and macrovascular inflammation associated with some of the semi-chronic changes in cardiopulmonary functioning observed in this study. Future cooking emission studies might consider longer-term assessments of these health outcomes over a greater number of repeated exposures to cooking events and/or completion of studies among populations with cardiovascular or pulmonary disease (e.g., high BP and asthma).

## Limitations and future work

The primary limitation to the present study is a relatively small sample size. A post-hoc power calculation with our outcome variables suggests a sample size of ~ 40 to see a statistical difference in our outcome variables, should one exist. Other limitations include the rather healthy environment (baseline PM_2.5_ values were extremely low under all conditions) and the use of very healthy subjects (with low BP values and baseline and FeNO values that are well below the ATS standard for airway disease). Due to the intense nature of the study, with four weeks of stay in a well-controlled residential apartment while measuring several environmental and physiologic variables, it is somewhat difficult to study larger groups of subjects. We do believe that this is worth further exploration, in both health and disease (particularly in hypertensive and asthmatic patients). Future work could also focus on geographic areas with higher baseline PM_2.5_, in order to observe a larger effect.

## Conclusion

This pilot study investigated the influence of air quality interventions with short-term exposure to PM_2.5_ on cardiopulmonary health indicators. Our findings indicate that PM_2.5_ levels increased significantly after cooking compared to pre-cooking levels in both the SCC and the ACC. Notably, the ACC resulted in a more substantial reduction in PM_2.5_ concentrations compared to the SCC. Such reductions may help curb short-term adverse cardiopulmonary outcomes by inhibiting the activation of the sympathetic nervous system and curbing the release of pro-inflammatory cytokines. These insights pave the way for further exploration and endorsement of HAP mitigation strategies in homes to counteract the detrimental impacts of particles generated by cooking.

## Methods

### Overview of study design

We employed a crossover design to investigate the influence of cooking-generated PM_2.5_ on cardiorespiratory function and the potential mitigation effects of an automated indoor air quality intervention on cooking emissions in two identical, simulated one-bedroom residential apartments at the Well Living Lab (WLL) in Rochester, MN, USA. The apartments had a total floor area of 32.6 m^2^ and featured a kitchen, living room, bedroom and bathroom. All methods were performed in accordance with the relevant guidelines and regulations. The study was reviewed and approved by the Mayo Clinic Institutional Review Board (#20–007908) and all subjects provided written informed consent prior to the study. Seven cohorts of two participants took part in the study (7 females, 6 males, 1 other, participant characteristics, Table 3) each staying within the apartment for four weeks. Details about the recruitment process, inclusion and exclusion criteria can be found in the Mayo Clinic IRB #20–007908. A constant airflow of 170 m^3^/h (a blend of external and recirculated air in a fixed ratio processed through MERV14 filters) was supplied to each unit, resulting in an air exchange frequency of two times per hour. A comprehensive characterization of the air distribution can be found in Liu et al. 2022^16^. This experimental setup featured air filtration levels higher than those found in most homes in the United States and exhibited high air tightness. A review of approximately 70,000 single-family homes reported a geometric mean (GM) natural infiltration rate of about 0.50 air changes per hour (ACH), with a geometric standard deviation (GSD) of roughly 2.0^45^. When compared to typical U.S. homes, the high air supply in our experimental setup would be balanced by the cleaner infiltration air entering an average home.

**Table 3:** Participant Characteristics.

	Mean ± SEM
Sex (% Female)	43
Age (Years)	37 ± 3
Height (Meters)	1.7 ± 0.02
Weight (Kg)	80 ± 5
BMI (Kg/m^2^)	27.2 ± 1.3

**Fig. 9:**
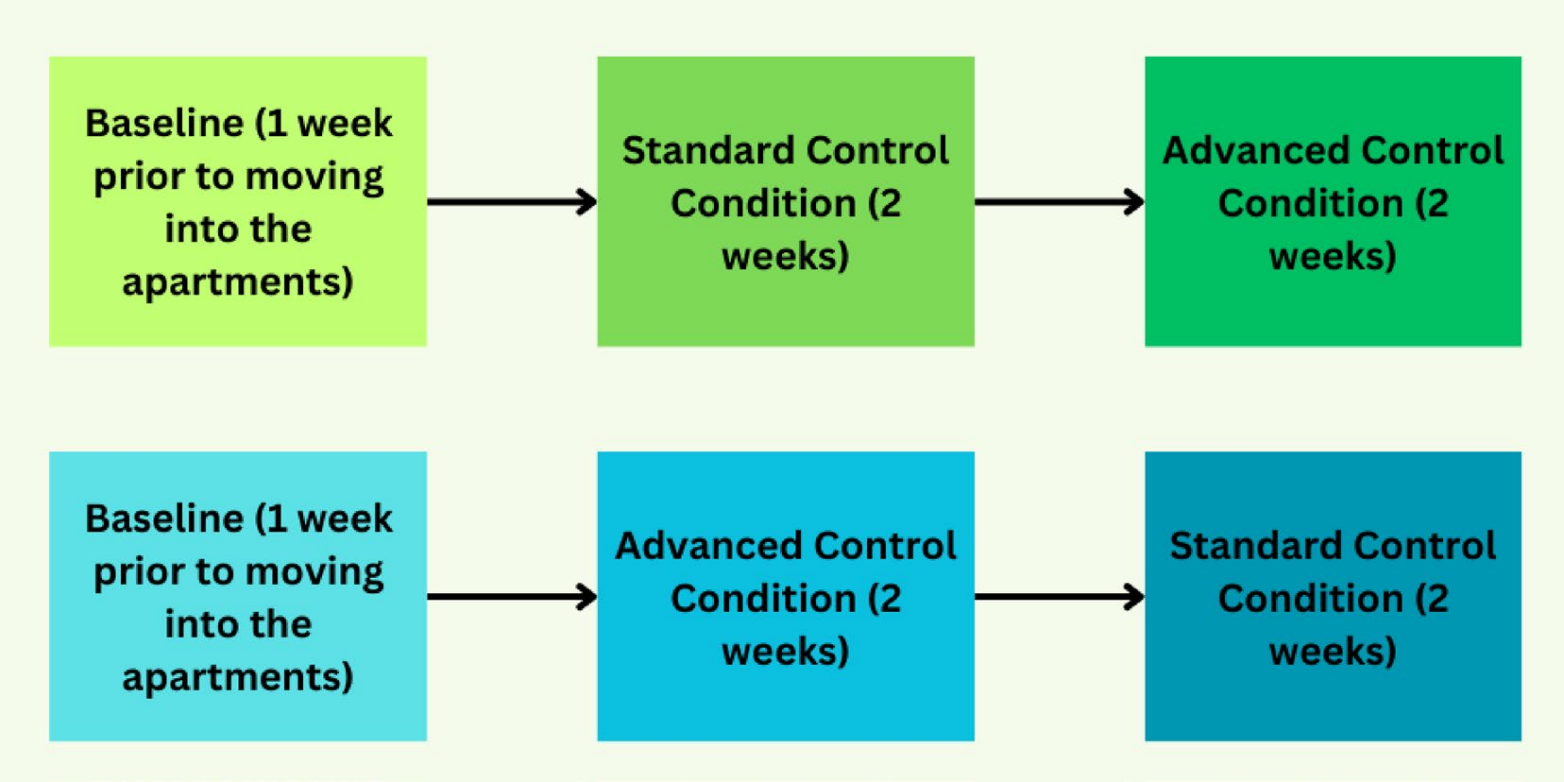
Sequence of experimental conditions (order was dependent on randomization).

Each week, participants relocated to the Well Living Lab (WLL) on Sundays and resided in the apartments until Friday evening. Participants had the option to depart on Friday evenings after completing all study-related activities and return on Sunday evenings. The experiment began with a one-week baseline measurement period conducted at each participant’s place of residence. Following this, the recruited participants were exposed to two experimental conditions in a randomized order. Each experimental condition lasted for two weeks and consisted of: (1) a SCC (usual HVAC operation in the residential environment); and (2) an ACC (indoor air quality interventions) (Fig. 9).

**Fig. 10:**
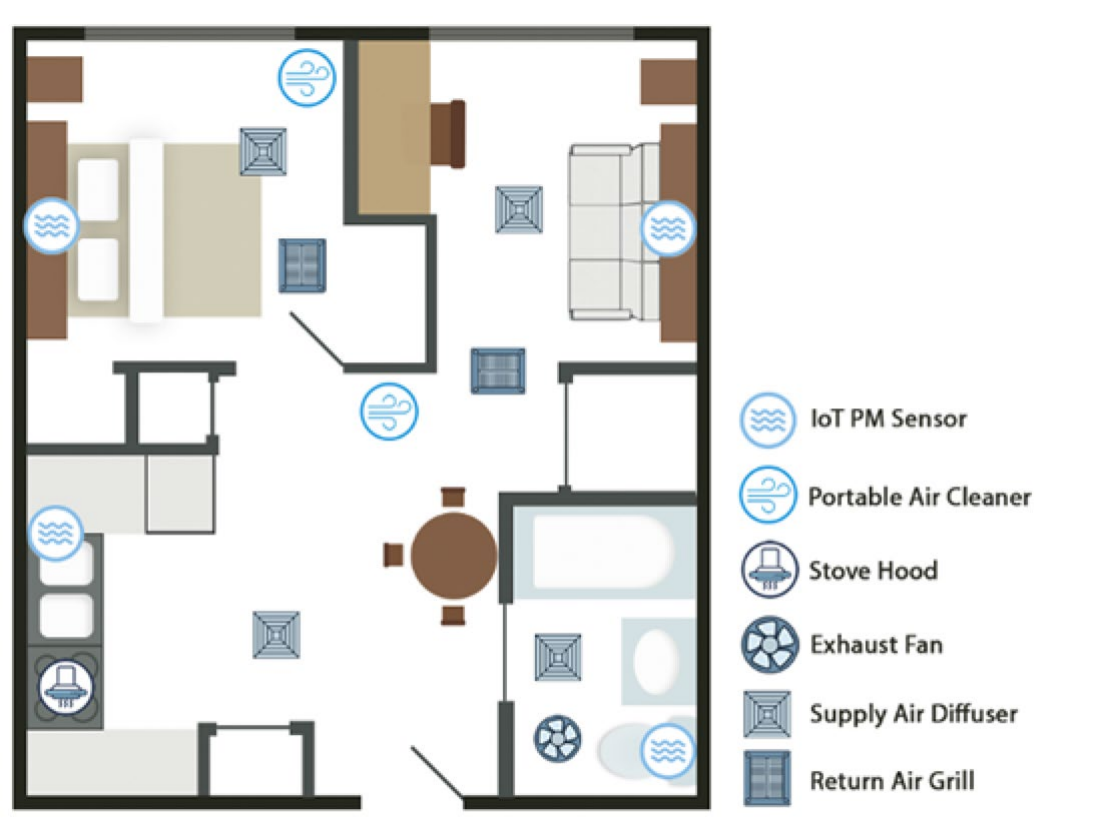
WLL residential apartment.

Participants were instructed to carry out a designated set of activities during the day. Briefly, these activities included cooking both breakfast and dinner using provided recipes and ingredients. More details can be found in Pantelic et al. 2022^46^. Additionally, the subjects performed tests to measure physiological indicators of cardiovascular function and airway inflammation, including BP, HR, HRV, and FeNO. Individual- and apartment-level PM measurements were continuously recorded throughout the study using an Internet of Things (IoT) PM sensor (PurpleAir PA-II, PurpleAir, Draper, UT) (Fig. 10). Physiological measurements were compared before and after cooking events for both experimental conditions.

### Experimental condition details

The SCC involved an overhead mixing air supply of recirculated and outdoor air. Return air grilles were located in the bedroom and living room, with a dedicated exhaust fan in the bathroom (see Fig. 10). The objective of the SCC was to maintain a consistent temperature set point by modulating the air supply temperature and keeping the air supply constant. Study participants were free to set their desired air temperature using a thermostat in the bedroom. The total air supply flow rate was consistently set at 170 m³/h, with balanced airflow among the diffusers. The outdoor air supply volume was designed to meet or exceed the ventilation standards set by the American Society of Heating, Refrigerating and Air-Conditioning Engineers (ASHRAE) 62.2–2019.2^47^. In addition to ventilation and recirculated air supply, the SCC featured a manually-operated stove hood and a bathroom exhaust fan.

The ACC retained the same air supply system as the SCC and introduced multiple automated air quality interventions operated based on measured PM_2.5_ levels. The ACC included a range hood in the kitchen that automatically operated based on PM_2.5_ readings from a sensor positioned adjacent to the exhaust channel. It also featured a PAC in the living room controlled by a PM_2.5_ sensor above the couch, a PAC in the bedroom with a corresponding PM_2.5_ sensor above the bed and a bathroom exhaust that operated based on PM_2.5_ readings in the bathroom (see Fig. 10). An indoor air quality intervention control algorithm activated each intervention when a PM_2.5_ reading in the corresponding control zone (Fig. 10) measured a PM_2.5_ concentration exceeding 15 µg/m³ and was deactivated after the average of three consecutive readings with a 1-minute frequency dropped below 6 µg/m³.

### Estimation of PM_2.5_ concentrations and environmental measurements

We calculated PM_2.5_ concentrations before and after cooking started by using measurements from the PM_2.5_ sensor in the kitchen (Fig. 10). Cooking events were identified using data from the dedicated stove circuit monitor (Sense Labs Inc., *Cambridge*,* Massachusetts*), which detected periods when the stove was in use, and PM_2.5_ measurements taken above the stove, which indicated when cooking emissions were occurring. We considered cooking to have taken place when the stove was turned on and PM_2.5_ levels rose above the baseline level^24^. We divided the exposure into two distinct intervals based on the start of cooking. The first interval, referred to as “before cooking”, spans the 30 min leading up to the physiological measurement (HR, HRV or BP). The times of the physiological measurements were recorded automatically by the devices used. The second interval, referred to as “after cooking started”, began with the onset of cooking and lasted until participants took another set of physiological measurements. We believe that considering these two time windows for PM_2.5_ concentration analysis aligns well with the biometric measurements recorded pre- and post-cooking, reflecting the impact of inhaled PM_2.5_ during the process.

### PM_2.5_ IoT sensor

Each apartment was equipped with a PM_2.5_ IoT sensor, specifically the PurpleAir PA-II model (PurpleAir, *Draper*,* UT*), as illustrated in Fig. 10. These sensors were affixed to a vertical surface at a height of 1.2 m and were designed to measure particle mass concentrations across six distinct particle sizes within a measurement range of 0 to 1000 µg/m^3^. After calibration, the measurement accuracy was ± 6 µg/m^3^ for readings below 100 µg/m^3^, and ± 10 µg/m^3^ for those ranging from 100 to 500 µg/m^3^. Data was recorded at 2-minute intervals throughout the study and then wirelessly transmitted to a cloud-based storage platform.

### Physiological outcome measures

We assessed various non-invasive physiological outcomes, including HR, time- and frequency-domain HRV, BP and FeNO at two distinct time points: (1) before the cooking activity began; and (2) within 30 min after the completion of cooking at various times throughout the participants’ stay.

HR and HRV were collected using the CorSense system (Elite HRV, *Asheville NC*) and further analyzed for the time- and frequency-domain components of HRV within the Kubios software package (Kubios Oy, *Kuopio*,* Finland)*. HR and HRV were collected during waking hours at four specific time points daily (Monday-Friday): (1) Immediately after the participant woke up, (2) after cooking breakfast, (3) before cooking dinner and (4) after cooking dinner. Resting HRV measurements were collected for approximately 10 min after the participant had rested for five minutes (stabilization period) with the participant seated and as motionless as possible using regular breathing.

Semi-continuous measurements of BP were collected before and after cooking using an ambulatory blood pressure monitor (OnTrak 90227, *Snoqualmie*,* WA*) on Tuesdays and Thursdays over the four-week period. In the morning, ambulatory BP was measured every 15 min during a two-hour period, from one hour before cooking to one hour after cooking for approximately 10 min after cooking breakfast. In the evening, ambulatory BP was also measured during a three-hour period, from 30 min before cleaning or cooking to 1.5 h after cooking dinner.

Measurements of concentrations FeNO were used to detect changes in airway inflammation as an index of pulmonary function. FeNO was collected using the NObreath FeNO monitor (Bedfont^®^ Scientific, *Kent*,* England*). Assessment of FeNO measurements were collected in triplicate *via* exhaled air. Participants collected these measurements during four scheduled periods, before and after cooking breakfast two days per week and before and after cooking dinner, two different days per week. An additional day included measurements collected before and after cooking, both breakfast and dinner on the same day.

### Statistical analysis

A matched-pairs analysis method using R (version 3.6.2) was used to examine differences between BP, FeNO, HRV and HR measurements before and after exposure to cooking-generated particles (“pre-” and “post-cooking”). Further, we employed population-averaged GEE to examine differences in BP, FeNO, HRV and HR changes within and between the SCC and the ACC. This method accounts for the correlation between repeated measurements and provides robust standard errors, making it suitable for our longitudinal data. Our GEE models (geepack, version 1.3-1.3) used an exchangeable within-participant covariance structure which assumed that physiological measurements taken from the same participant had a constant correlation and that the order of observations did not matter. We calculated covariance using the Eicker-Huber-White estimator, and we determined confidence intervals and p-values using a Gaussian approximation. An alpha of 0.05 was set to determine statistical significance.

Although the primary aim of the present study was not to assess a dose-response relationship between PM_2.5_ levels and acute changes in cardiopulmonary function, we conducted an initial analysis to explore associations between individual PM_2.5_ measurements and each physiological outcome variable across all time points, before cooking, after cooking and within SCC and ACC. Additionally, we performed a mixed general linear model (GLM) including day x participant x condition, as well as time (pre- and post-cooking), to assess differences between the two ventilation strategies. This analysis revealed a significant difference in PM_2.5_ levels between the ACC and the SCC.

To determine the statistical significance of the differences in PM_2.5_ concentrations observed in our experimental data, we employed the t-test, a commonly used statistical method for comparing the means of two groups. The t-test assesses whether the difference between group means is statistically significant, helping to determine whether the observed variation is likely due to chance or reflects a true underlying effect. We used a paired samples t-test to compare means from the same group at different time points. To validate the assumptions of the test, we confirmed that the data approximately followed a normal distribution using the Shapiro-Wilk test. For the independent samples t-test, we assessed the homogeneity of variances using Levene’s test. Additionally, we verified that the differences between paired observations were approximately normally distributed.

#### Data processing

PM_2.5_ data was collected continuously, recording each time data was saved. Data was filtered based on the periods when various physiological measurements took place. CorSense measurements of HRV and BP contained timestamps, as did the PM_2.5_ measurements. We created subgroups of PM_2.5_ data based on the collection time periods. We then grouped PM_2.5_ data with the same timestamps as the HRV measurements into two groups: (i) Before cooking—The period 30 min before cooking started; (ii) After cooking started that includes periods during cooking—the time between when cooking started and cooking ended and after cooking—the period 30 to 60 min after cooking ended.

## Data Availability

The datasets generated and/or analyzed during the current study are not publicly available but are available from the corresponding author upon reasonable request. Requests for data should be directed to Dr. Sara Aristizabal via email at sara.aristizabal@delos.com.
